# Correlation of Muscovite Sheet Mica on the Basis of Color, Apparent Optic Angle, and Absorption Spectrum*

**DOI:** 10.6028/jres.067A.033

**Published:** 1963-08-01

**Authors:** Stanley Ruthberg, Mary W. Barnes, Ralph H. Noyce

## Abstract

A detailed experimental study of color, apparent optic angle, and absorption spectrum (0.3 to 16*μ* of *λ*) indicates that there are basic chemical and structural differences in muscovite micas. Color identification is based upon spectrophotometer measurements. A method for quantitative evaluation of absorption band activity is defined so that single and multiple band intensities within the spectrum of an individual specimen and within spectra of different specimens can be compared. Three basic spectral types exist for the 0.3 to 1*μ λ* region which are comprised of various weak lines and absorption regions lying along the base of a deep absorption edge near 0.32*μ.* Of the three spectral types, one is associated with ruby micas, while the other two are associated with dark green and light green micas. The activities of numerous lines and bands are reported. It is shown that apparent optic axial angle is related to the position of the deep band edge except for a certain class of ruby specimens which show anomalous values. A definitive characterization of muscovite sheet is formulated in terms of the absorption coefficients at 0.44, 0.49, and 0.58*μ*. Direct associations are shown between certain lines in the visible λ range and certain bands in the near infrared, while the activities of a number of bands in the middle infrared are correlated to color.

## 1. Introduction

Muscovite mica, a complex crystalline mineral, is readily cleaved into thin elastic laminae which have excellent mechanical and electrical insulating properties. It occurs with large variations in physical appearance. Considerable effort has been expended to determine the range in values of its physical properties and to seek correlations between properties, particularly for those of technical significance to those properties by which the material is classified. At present, classification is by origin and color. The mineral, which occurs throughout the world, varies in color from green to pink. Further selection is based upon such properties as clarity, hardness, ease of splitting, and structural perfection [1, 2, 11].^3^ Numerous studies on dielectric constant and power factor [3–11], d-c resistivity [12, 13], electric strength [14, 15], gas evolution [16], thermal conductivity [17], thermal expansion [18], magnetic susceptibility [19, 20], and hardness [21], in addition to many others of chemical, mineralogical [22, 25] and infrared nature [27, 34–39] have failed to reveal any real correlation, and the material is still selected for technical use by arbitrary and empirical procedures which have incomplete or even doubtful quantitative significance.

A basic difficulty is that analytical chemical procedures have not been sensitive enough to differentiate muscovite sheet mica or provide a simple means of correlation with other properties, particularly in view of the complex of minor constituents which appear as isomorphous replacements in the mica structure. The position of a replacement cation in the mica structure is determined more by its size than its valence, although the valence state determines charge balance and hence relative abundance in the layers [25]. Normally, in addition to the major cationic components of K^+^, Al^+3^, and Si^+4^, a number of cations such as Na^+^, Ca^+2^, Fe^+2^, Fe+^3^, Mg+^2^, Ti^+4^, Mn+^2^, Mn+^3^, Li+, Cr+^3^, and V+^3^ occur in the same specimen. Total accuracy of component analysis is of the order of 0.1 percent, while the small amounts of material in isomorphous substitution can be in large relative error. Because of these errors and because calculation to unit formula requires a number of assumptions about the structure, analysis of this material is a special problem [23, 25]. Furthermore, calculation fails if an extraneous mineral is present in unknown proportions, defects are not considered, and the valence states of some cations are not always readily obtained; yet, the distinction of even exceptional specimens such as the rose micas depends upon subtle ratio and valency of small amounts of cations in isomorphous substitution [24]. Although success has been obtained in the classification and correlation of the many different dioctahedral potassium micas as based upon the tetrahedral-octahedral charge relationship [25], muscovite in this scheme is given as an end member of the trisilicic series where hardly any difference is indicated between pink muscovite and muscovite. Yet, the whole group of muscovites in this very narrow band of the trisilicic-tetrasilicic series must be differentiated.

The problem of characterization of muscovite sheet is thus subtle, and the required sensitivity for any differentiation must be found outside presently available chemical and structural analytical techniques. A study was therefore made of certain physical properties chosen because they might be indicative of chemical composition and structure and measurable with sufficient accuracy to allow quantitative differentiation. Moreover, the properties and procedures chosen permitted inspection of a large number of specimens. Careful interpretations of these properties within groups of muscovite mica may eventually help in understanding the crystal-chemical layer structure. More immediately they may correlate with technologically useful properties.

This paper, then, is not concerned with direct chemical methods but presents a detailed experimental study of the several selected properties. These are (1) color, (2) apparent optic axial angle, and (3) absorption spectrum from the near ultraviolet (300 m*μ*) to the middle infrared (16*μ*).

Although color is a major property by which muscovite sheet is sorted, it remains one of the least understood [11, 22, 24]. It should certainly be indicative of compositional variation. One study was made of the role of iron, titanium, and magnesium in the color of biotites [26], but no similar study of the muscovite group is known.

Winchell and Winchell [22] indicate that the apparent optic angle may be dependent upon the cations substituted into the charge layers, but values are available for only a few muscovites, phengites, and some mixings of these types. Little else is known.

Finally, it is reasonable for the absorption spectrum to be sensitive to electronic and molecular differences. Stubican and Roy [27] showed variation of infrared bands with gross isomorphous substitution, such as the change of muscovite to phengite, primarily for wavelengths longer than the first Si-O bands at 9 to 10/x. But, almost no spectral information is available from the near ultraviolet to the near infrared, nor has the large effort in the middle infrared been oriented toward characterization of natural mica sheet.

With the establishment of quantitative procedures and correlation of the properties studied, it is shown that muscovite sheet micas can be systematically differentiated in a manner related to morphological (crystal-chemical layer structure) differences. For example, the infused pink tinge that characterizes the ruby color class of muscovite is related to measurable anomalies, whereas, the other color varieties are fairly consistent within this descriptive behavioral scheme.

## 2. Specimens

Muscovite mica is normally distinguished by color, primarily as ruby (pinkish buff to cinnamon drab) or nonruby (brown to green), and by origin. Muscovite mica sheet is further classified visually according to an ASTM code which employs eleven categories, labeled V–l to V–11, of decreasing quality as based upon clarity, uniformity of color, structural perfection, and hardness [28]. Whereas classification under this system is subjective, expert inspectors obtain reasonably consistent and mutually agreeable results by constant intercomparison of samples and by the selection of suitable standard samples. Grading is also based upon the size of the usable area of the given quality. Specimens become rarer as quality and size of usable area increase.

The materials used in this study are described in table 1 according to color, origin, quality, grade, and amount obtained. Six representative origins are included. The items listed represent the colors of commercial sheet muscovite. Dimensions are shown in table 2. These had been classified by expert inspectors before being received by us from Defense Materials Service of the General Services Administration and from Committee F–l of ASTM. Specimens were cleavage plates ranging in thickness from 0.15 mm to 0.5 mm.

## 3. Experimental Methods

### 3.1 Color

Muscovite micas viewed in transmitted light are well known to range widely in hue and absorbance. Designation is normally in the broad category of ruby or nonruby; however, the title of ruby is more a classification than a description. Pinkish buff to cinnamon drab are generally termed ruby; brown to brownish olive are generally called rum; yellowish olive to green are called green; but as variations in color are continuous none of these designations is sharply defined, and the classification into ruby and nonruby often depends upon color differences so small as to be scarcely perceptible.

Actually, there are no formally accepted standards for color designation. Instead, an acceptable classification in the mica trade is formulated upon the experience and memory of the inspectors and upon constant intercomparison of representative samples of the color families. To decide color the inspector must take into account the thickness of the specimen, disregard inclusions, cloudy and stained areas, and make comparison to remembered samples.

The only definitive study on the color of mica was that of Judd [29] which established a color standard for ruby mica and was particularly applied to definition of borderline samples between ruby and nonruby. His separation into ruby and nonruby was based upon the lightness index (modified optical absorption coefficient) and hue index and “not on the hue index alone. That is, the grading of mica as ruby or nonruby according to trade practice departs considerably from grading purely on the basis of hue alone, and a specimen of light-colored mica can be considerably greener than a dark borderline specimen and still be graded ruby.” He further demonstrated that in spite of the problems encountered, the color designations of the inspectors are “surprisingly reliable and consistent.” In fact, the correlation between points on his lightness-hue index plane and the color grading into ruby or nonruby by a mica task committee was “nearly perfect.”

A number of factors were involved in the selection of an appropriate procedure for the present color study. One was the desire to consider the whole range of colors exhibited by muscovite sheet. Whereas Judd was particularly successful in the objective sorting of muscovite into ruby or nonruby, his results also showed that even the color category of “ruby” is very complex. A second factor was the consistency of trade-designated color categories. Third, was the fact that absorption spectra would be obtained for a range of wavelengths and would include the visible region, in which the spectrum is itself a measure of color. And lastly, to secure absorption spectra for the myriad of samples necessary to represent all variations in color and origin would have been an overwhelming task.

The procedure used combined the quantitative aspects of the spectrophotometer with a visual sorting operation similar to that outlined by Judd. A familiarity with all the materials of table 1 was first established. These lots were then further sorted into a convenient number of subgroups with visual standards as described below. Representative samples of subgroups were subjected to spectrophotometry. Thus, the quantitative effort was diminished without sacrificing clear-cut correlation of color to spectrum, coordination with trade-designated color descriptions was established, duplication of color measurements was avoided, and large numbers of samples were handled.

The items of table 1 represent the range of colors of commerical sheet muscovite, classified according to trade-accepted standards. Some had been classified as simply ruby or nonruby, each including a range of colors. Others had been further assigned to more definite color, e.g., the various categories of ruby in items 7 to 10, amber, brown, light green, and dark green.

#### a. Spectrophotometer

A double-beam recording spectrophotometer (Cary, Model 14M) was employed with a lead sulfide detector and a tungsten source. The wavelength scan speed was 2.5 m*μ*/sec and the chart speed was 2 in./min. The wavelength was not limited to the visible region but ranged from 0.3*μ* to 1*μ*. A quantitative measure of color can be derived from the visible region when integrated with the spectral response of the eye. Although the spectrophotometer is far too slow for sample sorting, when used as outlined above it provided excellent quantitative data.

#### b. Photometer

Often, samples differed in color only because of lightness or shade. Where all samples in a group were of one hue, differing only in lightness, a simple but quantitative photometric (Photovolt Corp., Model 520 M) examination with tungsten source and appropriate narrow band filter was preferred.

#### c. Visual

The procedure used for color grading was as follows:

1. About 25 specimens were drawn from the item considered. These were spread on a large white matte surface illuminated from above by “cool white” fluorescent lamps and arranged according to the ruby-rum-green color classification, with some departure as noted below. Those closest to the red end of the spectrum were placed at one end of a series, those closest to the green of the spectrum were placed at the other end of the series, and the intermediates were placed between. Account was taken of thickness after observation of the change in color accompanying cleavage of thick specimens into thinner plates.
2. Another batch was drawn from the item, examined in the same manner, and specimens fitted into the series. This was repeated until the series stabilized, i.e., until no further extremes or different colors appeared.
3. Standard samples were generated. A number of specimens were chosen at appropriate intervals along the series. These were cleaved to a thickness of 0.015 to 0.020 in. as measured with a direct- reading micrometer. The trade designated colors included in table 1 served as guideposts or fill-ins for this selection.
4. Rapid sorting then proceeded through the matching of each piece with the closest “standard,” compensating for thickness. Thus, subgroups were formed. The sorting continued until about a pound of the item was used. In general, dozens of specimens occupied each subgroup although extreme subgroups often had smaller populations.

Items 7 to 10 of table 1, as trade selected, progressed in color from pinkish buff toward brown. However, as has been noted [29], the color situation of ruby mica is much more complex. In fact the so- called ruby micas can exhibit a progression of colors from nearly colorless through yellowish grey to dark brown and even include specimens of a distinctly green cast. The one distinguishing feature is the inclination to red with increased thickness of sample. The large batch ruby items of table 1, i.e., 2 to 6, did indeed exhibit a wider range of colors than found in items 7 to 10. Some specimens were redder, others greener, and hence the subgroup color categories for ruby micas were chosen to extend beyond those of items 7 to 10. The designations chosen were pink, pink-tan, tan-green, and green, i.e., green cast. Pink and pink-tan overlapped item 7, normal good colored ruby, with pink including specimens more extreme in red. Tan corresponded to item 8, slightly dark. Tan-green overlapped items 9 and 10, while green included those specimens of distinctly green cast.

When the large batch nonruby item, 12, was sorted, a branching in color was observed. This item ranged from olive to green. Color did not simply transform from one to the other but shifted from olive to a distinct dark green on one branch and to a distinct light green on the other, i.e., there were specimens intermediate between olive and dark green and between olive and light green. Nonruby subgroups were chosen as amber, brown, olive, dark green, light green, and lime. The lime was a distinctly different green. An intermediate subgroup on one branch was dark green-olive. On the other branch intermediate subgroups were olive-white-green and white-green, where white-green still retained a smoky appearance as compared to light green.

Finally, one item, 15 (Canadian Clear Purdy), was treated individually. It appeared to be borderline between rum and green. This was distinguished by essentially one hue but variable shade. It was sorted by shade.

### 3.2. Apparent Optic Angle

Mica is optically biaxial with the negative acute bisectrix nearly normal to the basal cleavage or 001 plane. Thus, cleaved sections give a nearly centered interference figure [30]. The apparent optic angle, that is the emergent optic axial angle as viewed in air, is a property of the crystal sensitively dependent upon the magnitude of the three principal refractive indices and is an easily measured quantity. The equipment used by us is described elsewhere [31]. A precision of better than ±5′ arc is achieved for good specimens. Waviness of the mica surface, common especially among the lower ASTM groups, however, superimposes an additional uncertainty because of the uncertainty in orientation of the geometric normal to the cleavage plane.

### 3.3. Absorption Spectra

#### a. Near UV to Near IR (0.3 to 1*μ*)

There is little previous information for this part of the spectrum [32, 33]. Absorption spectra were obtained with the Cary 14M double-beam recording spectrophotometer as outlined under color.

#### b. Near IR to Middle IR (1 to 16*μ*)

Absorption spectra were obtained with a Beckman IR–4 recording spectrophotometer using standard slit schedule, unpolarized radiation, a NaCl prism, and a wavelength scan speed schedule of 1*μ*/min except in the 2 to 3.5*μ* region where the scan speed schedule was reduced to 0.2 or 0.08*μ*/min. The 1 to 16*μ* region is separated into two parts by the greatly increased absorption which begins with that of Si—O modes in the 8 to 10*μ* region and which continues in longer wavelengths. The spectra for wavelengths of 1 to 8*μ* were obtained with crystal plates of the same order of thickness as for the 0.3 to 1*μ* region, i.e., approximately 0.2 mm. The longer wavelength region was obtained with laminae of 5 to 12*μ* thickness. These 5 to 12*μ* thick laminar specimens were prepared under a low-power microscope with a scapel. With care this can be done after some practice and depends upon the excellent basal cleavage of the material. Such specimens are suitable up to a wavelength of 17*μ* after which detail is lost through the excessive absorption of more Si—O modes.

**Figure 1:**
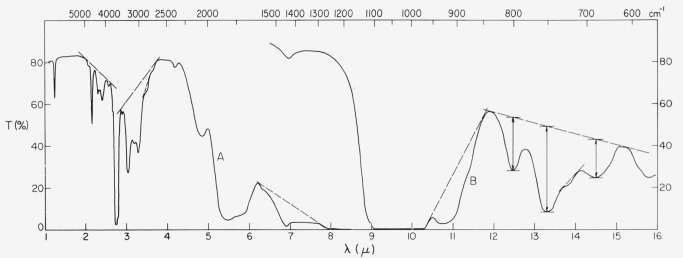
The 1 to 16μ λ absorption spectrum of a ruby muscovite specimen (item 2, table 1). Thickness of 0.201 mm.Thickness of 8*μ*.

**Figure 2:**
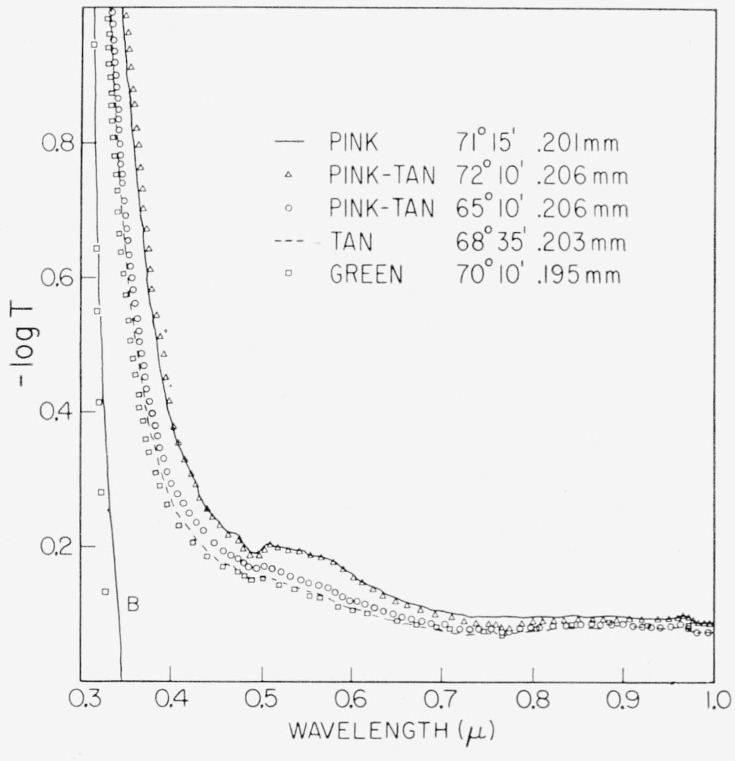
The 0.3 to 1μ *λ* absorption spectra of specimens representative of the color subgroups of ruby mica. Color subgroup, apparent optic axial angle, and thickness are listed for each specimen. Curve B is—log *T*=1 to 2. Specimens from item 2, table 1 (V–2 ruby India).

A typical spectral trace is shown in figure 1. The presence of most of these bands has been observed before [27, 34–39]. A few have been given definite assignment to O—H, Si—O, and A1—OH vibrations, some have been given a general assignment such as to Si—O—(Al) modes, but most are of unknown origin. Examination of the spectra of numerous representative samples indicated that differences appeared as small changes within multiple band regions such as the 3 to 4*μ*, and 12 to 15*μ* wavelength regions of the infrared (see fig. 1) and as the 0.5 to 0.6*μ* wavelength region of the visible (see fig. 2).

The determination of absorption band intensity is not an established procedure; thus, there is the task of finding a scheme appropriate for the situation. The need is to compare the intensities of the many absorption bands of one individual specimen and to compare the band intensities of one specimen to those of other specimens.

The variation in specimen thickness had to be accounted for. It was convenient to use the absorption coefficient, related to the transmission by Lambert’s law that *T=ex*p [− *α_λ_t*]. *T* is the ratio of the transmitted to incident radiant intensities, *I/I*_0_, *α*_λ_ the absorption coefficient at a given wavelength, and *t* the thickness of the specimen. This is for internal absorption. The values obtained with the transmission spectrophotometer must be corrected for surface effects. When a beam passes through an absorbing sheet, it suffers reflections at both surfaces. If the beam is sufficiently attenuated in the crystal plate, multiple internal reflections need not be considered. This is essentially true as long as no interference patterns are observed in the spectrophotometer trace. Then one finds that

$$-\alpha_{\lambda }t=\ln\;T^{\prime }-\ln\;{\left(1-r\right)}^{2}$$

where *T*′ is the machine value of the transmission and *r* is the reflection coefficient. The internal transmission is *T=T*′/(1 *− r*)^2^. Evaluation of *α*_λ_ and of *r* may be obtained from two splittings of different thickness from the same specimen, after which the spectral transmissions may be compared for any thicknesses.

When the wavelength corresponds to the peak of an absorption band, the value of *α* so obtained is the total absorption coefficient at resonance, for it is the sum of the absorptions due to the background and to the resonance condition itself. Thus, it is necessary to separate out the background contribution. This was attempted in these multiple band regions with the aid of base lines placed as indicated by the dashed lines in figure 1. A resonance absorp- tion coefficient (below background), *α*_λ_(*R*), is defined as

$$\alpha_{\lambda }(R)=-\frac{1}{t}\ln\;(T_{R}/T_{b})$$

where *T_R_* is the transmission at the resonance band center and *T_b_* is at the hypothetical base line at the same λ (see fig. 1). Note that

$$\frac{T_{R}}{T_{b}}=\frac{I_{R}/I_{0}}{I/I_{0}}=\frac{I_{R}}{I}$$

where *I_R_* is now compared not to the beam intensity before it strikes the crystal but to that which would be transmitted through the crystal if no resonance were present.

The position of the base line for a band had to be established. Whereas, the base line concept has been used before for a well defined, isolated band [27, 40], the present situation is certainly not so simple. To locate the base line, absorption spectra were obtained for samples of different thicknesses cleaved from the same original specimen. Trials of a base line location for a given band were made, and the resonance absorption coefficient was computed from each of the sample spectra. That orientation was chosen which yielded the least spread in the value of the coefficient for the particular band. This was repeated with a number of representative specimens. The base line for a multiple band region was so fixed that the coefficient for each of the included bands was so satisfied. The same procedure was extended to the shorter wavelengths of 0.3 to 1*μ.* Accuracy was usually obtained to within a few percent.

The *α’s* so calculated are, of course, an approximate measure of absorption band intensity but the interest here is on relative behavior. Additional errors beyond that of the procedure itself, namely those from determination of *t* and *T*, can be considerable. Although the *t* of the thicker plates was determined within 1 percent, the value for the 5–12*μ* thick specimens could be in error by 20 percent because of variations due to cleavage faults. Values for faint absorption bands can be in error because of small differences between *T_R_* and *T_b_.* The consistency actually obtained can be seen in the data below.

Empirically consistent relationships between some of the intrinsic band intensities, as herein defined, and the color data have been found. It is felt that this correlation of itself justifies the chosen procedure.

## 4. Results

### 4.1. Absorption Spectra, 0.3 to 1*μ*, and Color

Absorption spectra obtained by other workers had indicated the 0.3 to 1*μ* wavelength region to be relatively featureless [32, 33], but closer examination of the range shows it to be populated by a number of weak bands of absorption. These lie along the base of a very deep absorption edge and vary from sample to sample. The activities of these bands are shown to be directly associated with color.

**Figure 3:**
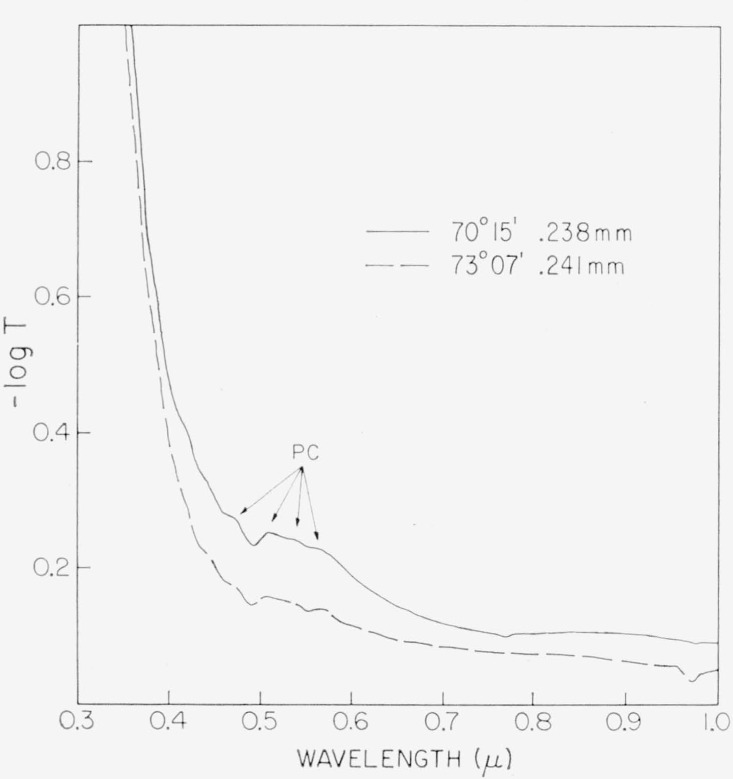
Internal detail of the 0.47 to 0.6/μ spectral structure for two representative ruby specimens. Maxima are indicated at 0.4725, 0.505, 0.535, and ~.57*μ*. These are labeled as PC for pink correlation. Material—item 1, table 1(V−1 ruby India).

This region is represented in figures 2 and 3 for ruby micas. Representative samples were selected for each of the color subgroups from item 2 of table 1, a V–2 India mica. A representative spectrum for each subgroup is displayed in figure 2 except the tan-green which was omitted for clarity. Spectra for two specimens of pink-tan of different values of apparent optic angle are included.

Several features may be observed in these spectra. First is the wavelength shift with the color subgrouping of the deep band edge near 0.32*μ*. Popper [32] found this absorption to extend to wavelengths shorter than 0.25*μ*. Then, there is a characteristic weak spectral structure at 0.472 to 0.6*μ* which consists of a band at 0.472*μ* and a broad absorption at 0.51 to 0.6*μ*. The fine detail of this broad absorption region is better seen in figure 3 for two specimens taken from item 1, a V–1 India mica. Three overlapping bands of absorption are indicated at 0.505, 0.535, and approximately 0.57*μ*. There are, in addition, two faint bands of absorption at 0.417 and 0.442*μ* and a broad response between 0.77 and 0.97*μ*.

In general, the progression along the color series for ruby materials from pink toward green is accompanied, as shown, by an increase in transparency, a shift in the band edge and, particularly, a diminution of the 0.47 to 0.6*μ* spectral structure.

The break in magnitude of the 0.47 to 0.6μ ab sorption between the two pink-tans of figure 2, otherwise distinguished by value of apparent optic angle, should be noted.

**Figure 4:**
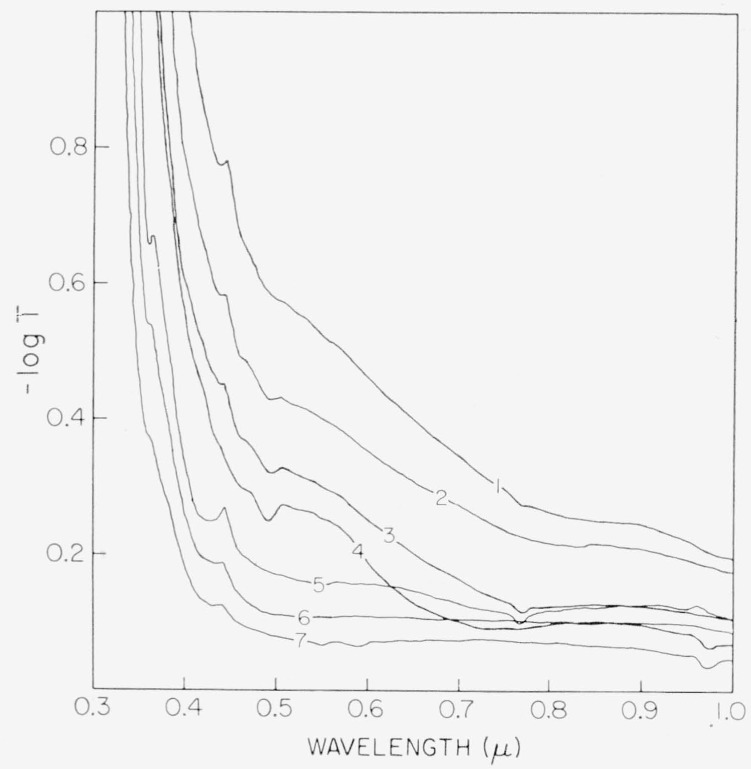
The 0.3 to 1μ *λ* absorption spectra for specimens representative of the color subgroups of rum and green micas with a pink ruby spectrum included for comparison. Color subgroup, origin, quality, and specimen thickness corresponding to the numbered spectra are: (1)—*dark green*, Tanganyika, V–l, 0.262 mm, (2)—*olive*, U.S., V–4, 0.210 mm, (3)—*amber*, Brazil, V–l, 0.226 mm, (4)—*pink ruby*, Brazil, V-l, 0.325 mm, (5)—*green-white*, U.S., V–4, 0.315 mm, (6)—*lime*, India, V–l, 0.248 mm, (7)—*light green*, India, V–l, 0.246 mm.

**Figure 5:**
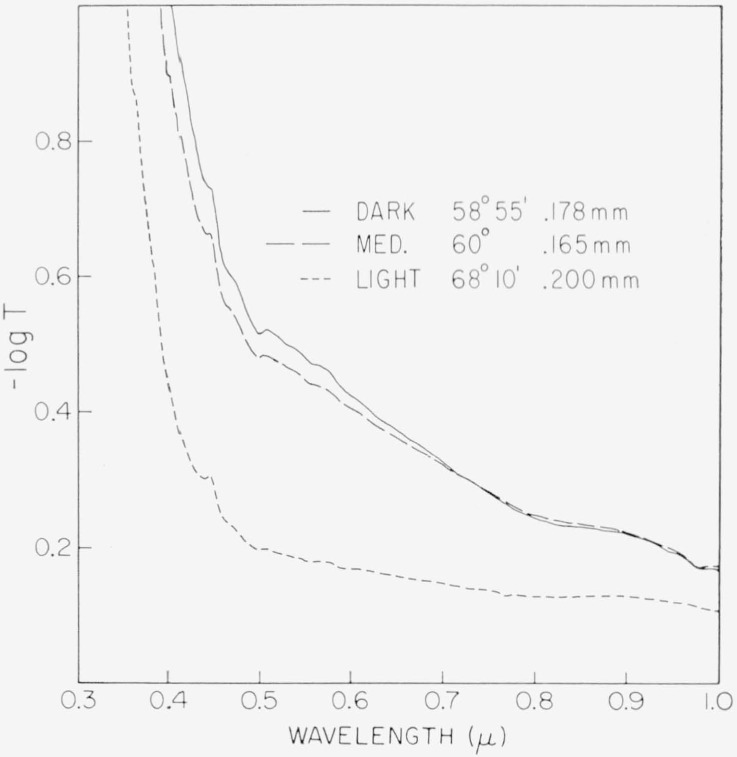
The 0.3 to 1 μ *λ* absorption spectra for specimens representative of the three shades of dark, medium, and light of a borderline rum-green mica (Canadian Clear Purdy, item 15—table 1). Apparent optic axial angle and specimen thicknesses are listed.

The 0.3 to 1 *μ* wavelength absorption spectra for rum and green micas are summarized in figures 4 and 5 with spectra for representative specimens from a number of color subgroups. Colors included are amber, olive, dark green, white-green, light green, and lime in figure 4 along with several shades of the borderline rum-green (Canadian Clear Purdy) in figure 5. The spectrum of a pink ruby was inserted into figure 4 for comparison.

The spectral details characteristic of nonruby micas are the relatively sharp and “strong” absorptions at 0.36*μ* and at 0.4425*μ.* The green micas have little of the 0.47 to 0.6*μ* absorption structure; whereas, the amber and olive associated spectra include moderate 0.47 to 0.6*μ* absorption. The difference between the dark green and light green spectra is the very much increased general absorption of the dark green with a long, almost straight rise from about 0.8 to 0.5*μ*.

Being an integrated response, the colors would not in themselves indicate details of the corresponding spectra, nor would the weak and sharp lines contribute much to the resultant color; thus, what is interesting in these results is that the color of muscovite is associated not with one spectral type but with spectra containing a number of distinctly different details. It is seen in the examples of figures 2 to 5 that all of these patterns may be described in terms of three basic spectral types and their combinations. One type is associated with ruby micas (curve 4, fig. 4). The main feature is the 0.47 to 0.6*μ* absorption structure which indicates the degree of pinkness. The other two are represented by the extreme curves of the greens, i.e., dark green (curve 1, fig. 4) and by light green (curve 7, fig. 4). The attenuation of the pink correlated region of 0.47 to 0.6*μ* transforms pink ruby to green ruby category, and its superposition upon the green spectral types is associated with olives, ambers, etc.

### 4.2. Absorption Spectra, 1 to 8*μ*, and Color

**Figure 6:**
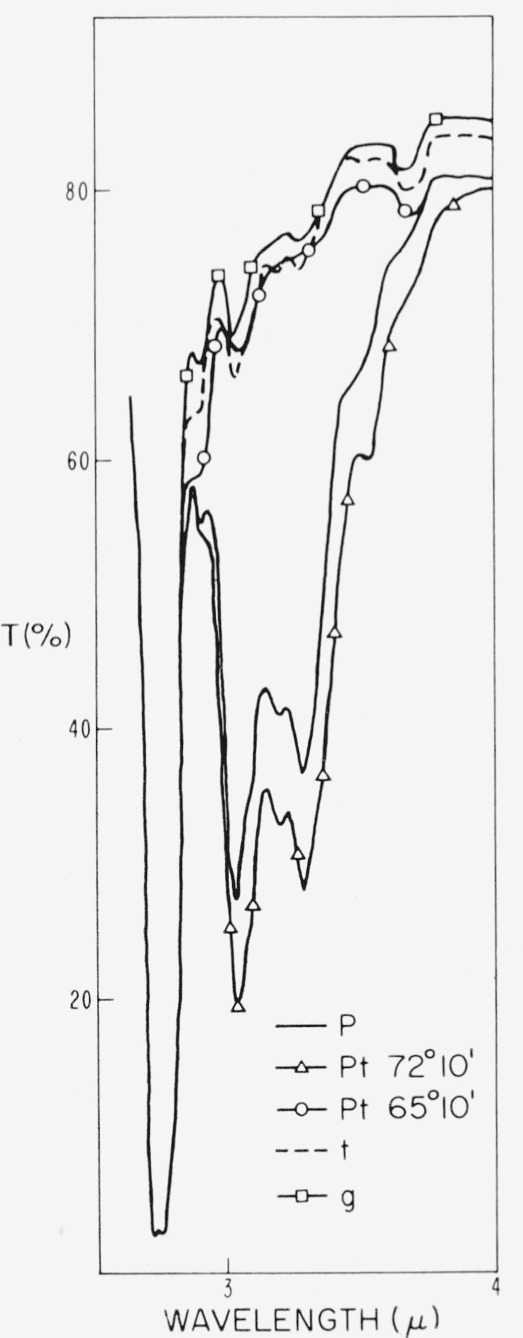
The 2.5 to 4 μ *λ* absorption spectra for specimens representative of the color subgroups of ruby mica. Specimens were the same as represented in figure 2.

**Figure 7:**
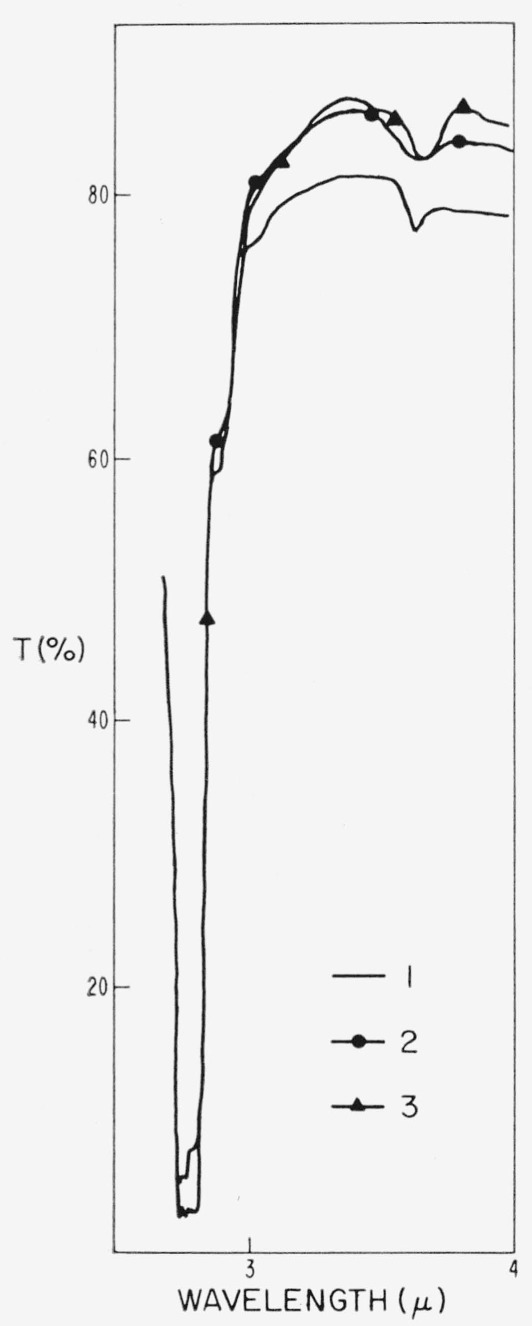
Representative nonruby absorption spectra, 2.5 to 4μ λ. *dark green*, Tanganyika, 0.256 mm thick (see curve 1, fig. 4)*amber*, Brazil, 0.266 mm (see curve 3, fig. 4)*green white*, U.S., 0.304 mm (see curve 5, fig. 4)

There are a number of prominent bands in this part of the spectrum as can be seen in figure 1. Some of these have not been reported before, some have appeared occasionally in previously reported spectra, and few have definite assignment. Most interesting from the present viewpoint is the absorption multiplet at 3 to 3.7*μ.* It appeared to be an anomaly to Sutherland et al. [37], since their muscovite spectra were normally devoid of the structure. On the other hand, it appears in some of the earliest spectra of ruby muscovites [34]. Matossi and Bronder [35] attempted assigning one of these bands, i.e., 3.05*μ* to an overtone of H—O—H deformation. In the present investigation, however, the component bands of this multiplet have always shown a common activity. Thus, any assignment of band origin must take this into consideration. Whereas, Sutherland et al., reported that they found no connection of this multiplet to color, the present group of samples show it to be associated with color in a most direct way. Examples of this 3 to 4*μ* absorption are shown in figures 6 and 7 for the specimens whose 0.3 to 1*μ* spectra appear in figures 2 and 4. This spectral structure has been found only in ruby specimens. It is particularly active in samples from the pink end of the color series but decreases to much smaller absorption values in those ruby specimens further along the color series, just as did the magnitude of the visible pink-correlated absorption region at 0.47 to 0.6*μ*. Other quantitative data obtained from many specimens are given in section 4.3.

Sutherland et al., also found the 7*μ* band to be companion to the 3.05*μ* band. This is verified in an appendix to the present paper. They further found the 2.3 to 2.4*μ* spectral structure to have a polarization effect in the same direction as the 2.8*μ* OH band and in the opposite direction as the sharp 2.2*μ* band. It is shown in the appendix that this 2.3 to 2.4*μ* structure also has some behavior related to color but in less straightforward fashion.

### 4.3. Absorption Coefficient Representation, 0.3 to 8*μ*

In section 4.2 it was shown how colors are related to absorption spectra. These in turn are more directly indicative of crystal-chemical differences. The details of the spectra can be quantitatively analyzed in terms of absorption coefficients.

Of immediate interest are the two absorption regions associated with the pinkness of specimens, the 0.47 to 0.6*μ* visable absorption structure and the 3 to 3.7*μ* infrared absorption structure.

**Figure 8:**
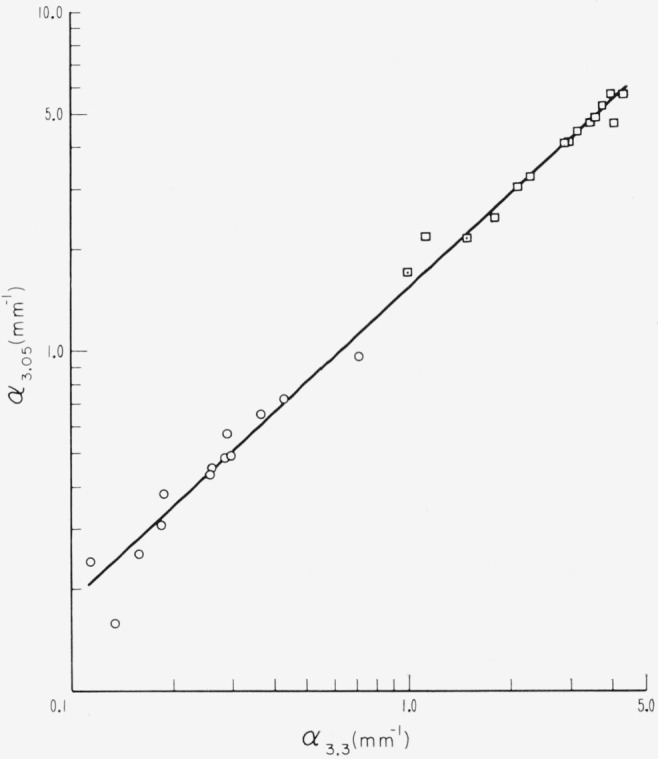
The activities of the two strong absorption bands at 3.05 and 3.3μ *λ* found in ruby muscovite micas as measured by value of absorption coefficient, α. Data points with values >1mm^−1^ are defined as representative of pink ruby (□). Points with values <1mm^−1^ are defined as representative of green ruby (○).

Of these two regions, the 3 to 3.7*μ* absorption is strongly associated with the redness of specimens, for it has only been found in ruby specimens and then with large absorption for pink rubies or with diminished values for the rest of the ruby subgroups. This situation is shown in figure 8 by data obtained for the two strong bands at 3.05 and at 3.3*μ* of λ. Data obtained from specimens from the pink end of the ruby color series, i.e., from pink and from pink- tan samples, usually appeared in the region for which values of absorption coefficient were greater than 1 mm^−1^ at both 3.05 and 3.3*μ*. In the case of pink- tans, this was usually true for those specimens with apparent optic angle ≥69°. On the other hand, a few specimens from the visually selected pink color end of the series gave coefficients slightly less than 1 mm^−1^. Points representative of all the other ruby subgroups are in the region of small values of *a.* Considering the complications inherent in the visual sorting of ruby muscovites into color subgroups because of ramifications of shade and other colorations than buff, this correlation is unusually strong. Reexamination of the specimens confirmed the strong inclination to red in those in the upper values of *α* at 3.05 and 3.3*μ.* It may be further noted that the density of points is relatively small in the vicinity of *α*= 1 mm^−1^.

Because of this strong correlation between the magnitudes of the absorption coefficients of the 3.05 and 3.3*μ* bands and the pinkness of ruby muscovite specimens, we hereafter designate rubies as being of two classes, i.e., either pink or green, as dependent upon the domain into which the values of *α*_3.05_ fall, with the division set at *α*=1mm^−1^. The data points are so coded in figure 8.

It is noted that the absorption coefficient at 3.05*μ* (*α*_3.05_) is greater than that at 3.3*μ* (*α*_3.3_) in specimens for which the values are small. The intensities of the two became more nearly equal in specimens for which the values are large.

**Figure 9:**
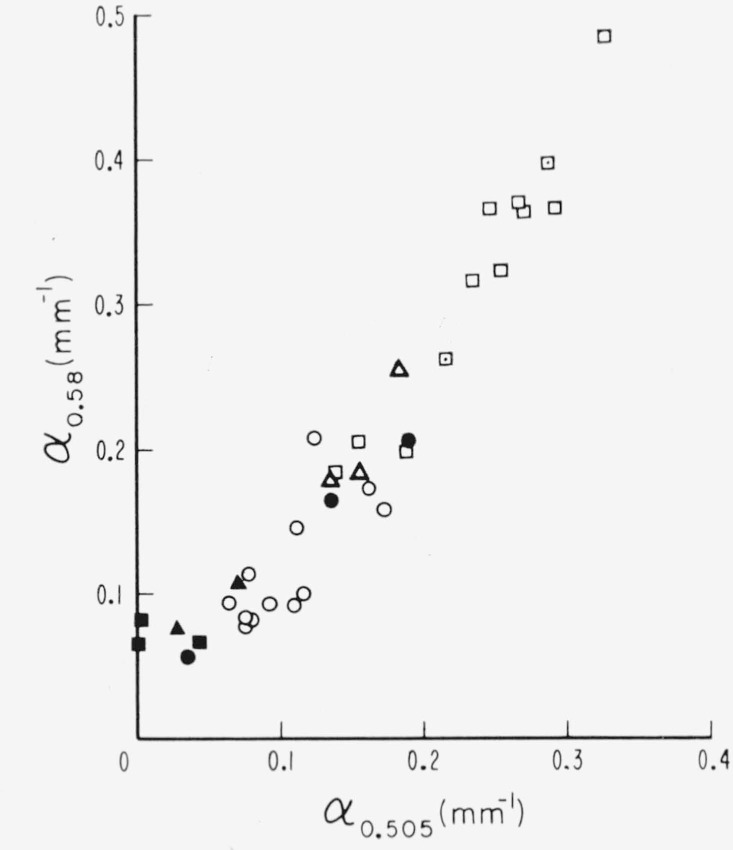
The activities of the 0.505 and 0.58μ *λ* lines of the visible 047 to 0.6 μ absorption structure as measured by values of absorption coefficient, α. □—pink ruby, ⊡—intermediate pink ruby, ○—green ruby, △—rum, ▲—dark green, ■—pale green, ●—rum-green (Canadian Clear Purdy).

Of the individual absorption lines in the 0.47 to 0.6*μ* visible absorption region, the two most useful are those at 0.505*μ* and 0.58*μ*. It was observable in figures 2 to 4, e.g., curves 5 and 7 of figure 4, that when the amount of absorption in this region is small, the 0.58*μ* absorption can be present without that at 0.505*μ*. Many specimens, e.g., curve 6 of figure 4, had neither absorption. The intensities of these two lines and their relationship are shown in figure 9 with data drawn from many specimens representating the various color subgroups. Points for which both absorption coefficients were zero were excluded. Points are here coded according to color but, for facility, with some contraction in that several subgroups have been combined. The pale green colors of light green, lime, and white-green are now included in one category. The dark greens are considered separately. The ambers and olives are lumped together as rums. The rubies are considered only as pink or green according to value of *α*_3.05_. As can be seen, *α*_0.505_ and *α*_0_._58_ are least for light greens and greatest for pink rubies. Ruin and intermediate green colors, i.e., ambers, olives, etc., yield intermediate values of *α*. Again, it can be seen that the dependence of the 0.58*μ* line is a little different from that of the 0.505*μ* line. It is seen by comparision of figures 8 and 9 that the values of *α*_3.05_ and *α*_3.3_ are greater than those of *α*_0.505_ and *α*_0.58_.

**Figure 10:**
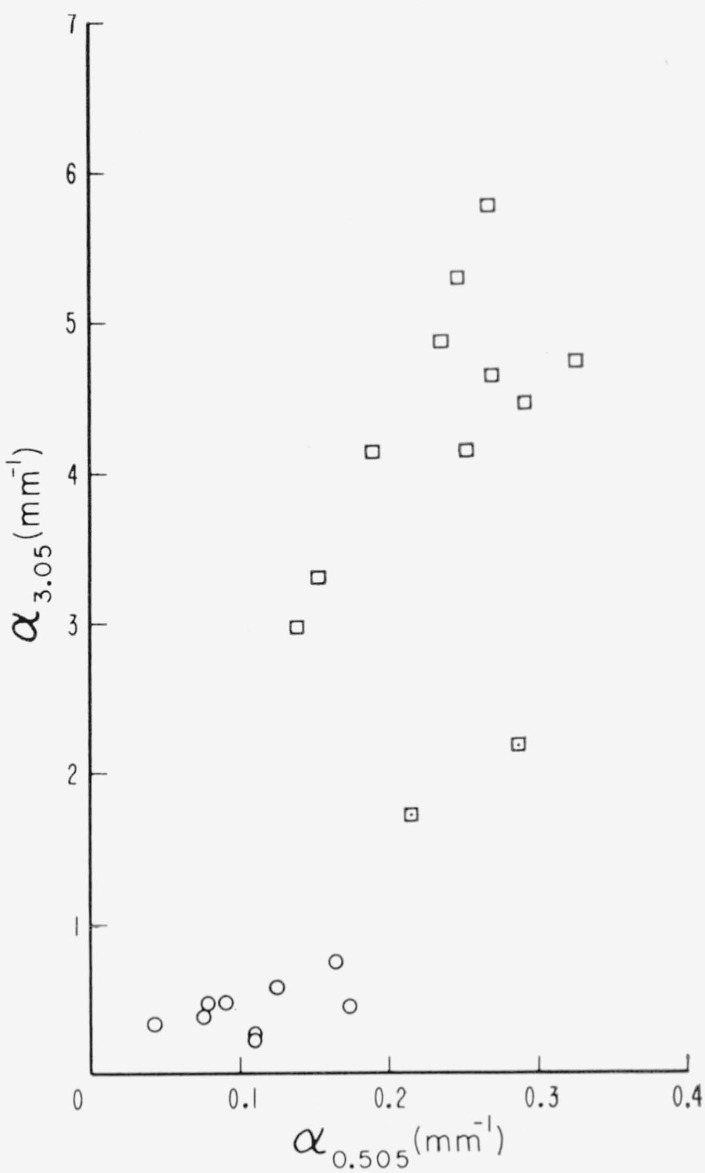
The relationship of the visible pink associated region to the infrared pink associated region; α*_3.05_* versus α*_0.505_*. □—pink ruby, ○—green ruby, ⊡—two specimens with intermediate values of α_3.05_.

Then, the relationship between the 0.47 to 0.6*μ* and 3 to 3.7*μ* regions is found in figure 10 where the visible wavelength region is represented by the 0.505*μ* line and the infrared region by the 3.05*μ* band. It is evident here that most points fall into one of two regions, i.e., low values of α_3.05_ representative of green ruby muscovite and large values of *α*_3.05_ representative of pink ruby. The region of the green ruby specimens is bounded by a value of *α*_0.505_ near 0.2 mm^−1^. Two intermediate values of *α*_3.05_ were observed. The data for these two specimens have been additionally coded in this figure and in the others for distinction.

**Figure 11:**
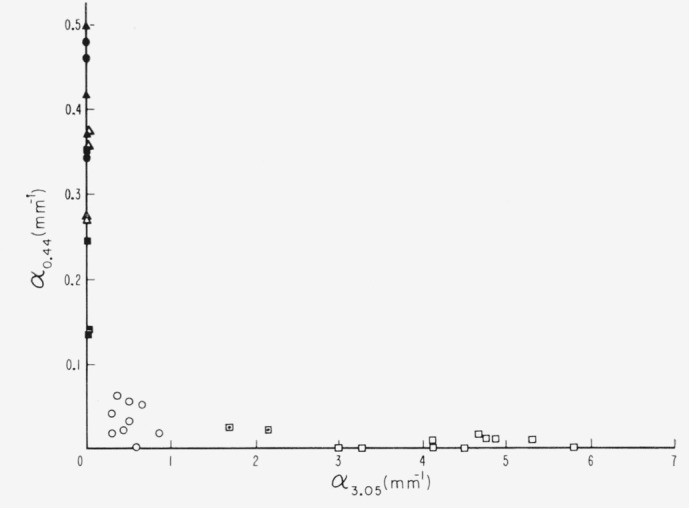
The relationship of the green associated 0.44μ line to the pink associated infrared region; α*_0.44_* versus α*_3.05_*. □—pink ruby, ⊡—intermediate α_3.05_, ○—green ruby, △—rum, ■—pale green, ▲—dark green, ●—rum-green.

There is one more pertinent relationship. This involves the 0.44*μ* line, the existence of which is an identifying feature of green micas. It is almost always vestigial in the pink ruby muscovites. The relationship between this line and the 3.05*μ* band is shown in figure 11. It is clear that these two absorptions are essentially mutually exclusive; that is, whenever *α*_0.44_ is relatively large, *α*_3.05_ has zero value; whenever *α*_3.05_ is relatively large, *α*_0 44_ has a near zero value. The progression from pink to green ruby muscovite is usually accompanied by an increase of *α*_0.44._ The nonruby muscovites observed have never had more than the barest value of *α*_3.05_.

It is now noted that the two pink ruby specimens represented in figure 10 by intermediate values of *α*_3.05_ are distinguished by values of *α*_3.44_ comparable to those of green ruby muscovite.

**Figure 12:**
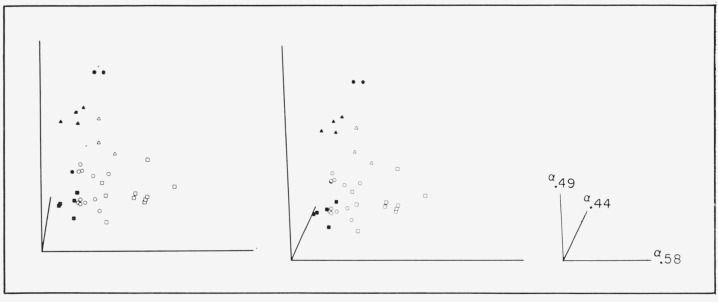
Stereo representation of the 3-coordinate characterization of muscovite sheet with absorption coefficients at wavelengths of 0.44, 0.49, and 0.58μ. □—pink ruby, ○—green ruby, △—rum, ■—pale green, ▲—dark green, ●—rum-green.

**Figure 13:**
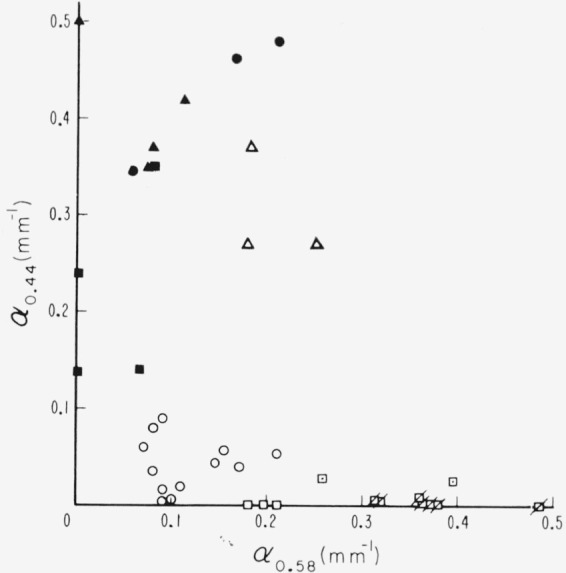
Projection of the 3-coordinate data space of figure 12 onto the [α*_0.44_*, α*_0.58_*] plane (view of the 3-coordinate space from above). □—pink ruby, ⊡—2 rubies of intermediate α_3.05_, ○—green ruby, △—rum. ■—pale green, ▲ —dark green, ●—rum-green, ⊠—pink ruby specimens for which α_0.58_ >0.2^+^mm^−1^.

**Figure 14:**
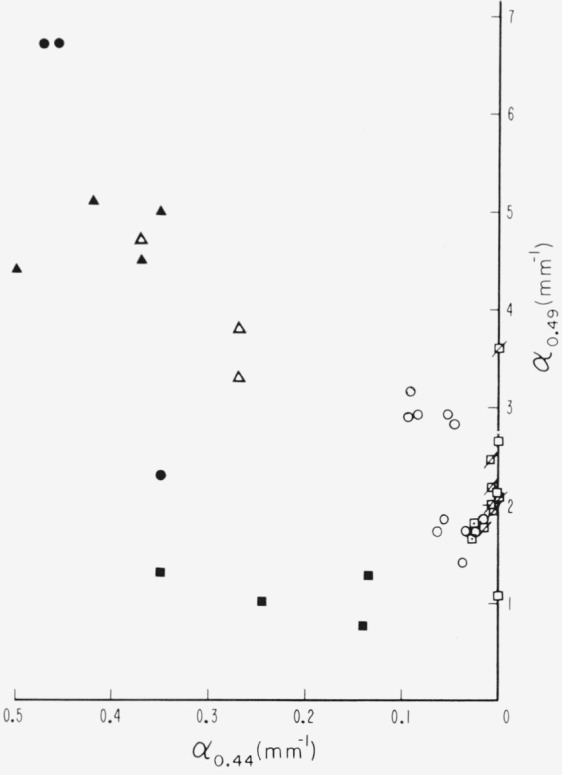
Projection of the 3-coordinate data space of figure 12 onto the [α*_0.49_*, α*_0.44_*] plane (view of the 3–d space from the left). See figure 13 for code.

With these quantitative results it is possible to describe muscovite sheet mica in a reasonably definitive way by a 3-space of absorption coefficients, *α*_0.44_, *α*_0.49_, and *α*_0.58_ The *α*_0.44_ applies to the green correlated line at 0.44*μ*. The *α*_0.508_ is chosen instead of *α*_0.505_ to represent the visible pink correlated region because it allows a little more differentiation of specimens possessing small values, as indicated by figure 9. The *α*_0.49_ is the total absorption coefficient, i.e., $-\frac{1}{t}\ln\;T$, for the minimum point of the absorption window between the 0.4725 and 0.505*μ* lines (see the minimum in the −log *T* representation of figs. 2 to 4), uncorrected for reflection. This is chosen to represent the general level of the absorption curve and, hence, the position of the band edge. Such characterization is shown by the stereo representation in figure 12 of the 3 dimensional data space so generated. Projections of the data onto the coordinate planes are given by figures 13 and 14. Figure 13 is the projection downward onto the [*α*_0.44_, *α*_0.58_] plane and figure 14 represents the view into the 3–*d* data space from the left of the stereo presentation.

The data for all the ruby materials fall close to the [*α*_0.49_, *α*_0.58_] plane, as *α*_0.44_ is small, while *α*_0.49_ ranges from about 1 to 3.5 mm^−1^. This is best seen in figure 14. As seen in figure 13, the pink rubies have large values of *α*_0.58_ but values for *α*_0.44_ near zero (except the two pink specimens with medium values of *α*_3.05_). The green ruby specimens are in the region of small values of *α*_0.58_ and *α*_0.44_. Data for the pale green specimens are located in figure 14 at small values of *α*_0.49_ of the order of 1 mm^−1^, and so are relatively transparent, but the 0.44*μ* λ line is comparatively intense. Pale greens may also have small values of *α*_0.58_ (see fig. 13). As the series progresses from pale green to dark green, *α*_0.44_ and *α*_0.49_ increase (see fig. 14). The rum micas are represented by points intermediate in all coordinates. In fact for these data the ruby, pale green, dark green, and rum-green groups generate a rough parabola on the [*α*_04_, *α*_0.58_] plane with the rum colored specimens located in the hollow.

Thus, it appears that these three coordinates provide a detailed basis for discrimination of muscovite micas and can be used to classify samples on a numerical basis.

### 4.4. Apparent Optic Axial Angle

**Figure 15:**
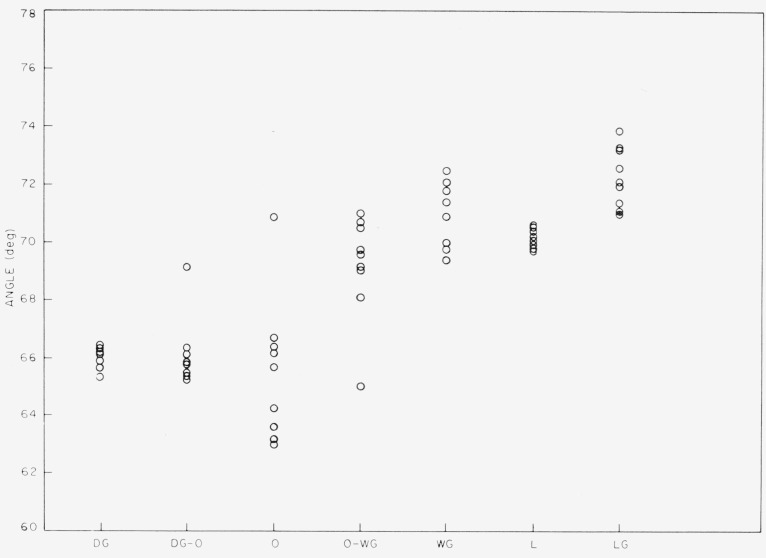
The variation of apparent optic axial angle versus nonruby color subgroup. V–4 nonruby Domestic, item 12—Table 1. Both color series branching off olive are represented, i.e.—dark green to olive, and light green to olive.

The apparent optic axial angle was determined for numerous specimens of each color subgroup in order to investigate the connection between angle and color. No significant correlation was found for the ruby micas, but a correlation did appear for the nonruby groups. In figure 15, results are shown for specimens of V–4 nonruby, item 12 of table 1, sorted according to color subgroup. The two color series branching off from olive are included, dark green being the end member of one branch and light green that of the other.

**Figure 16:**
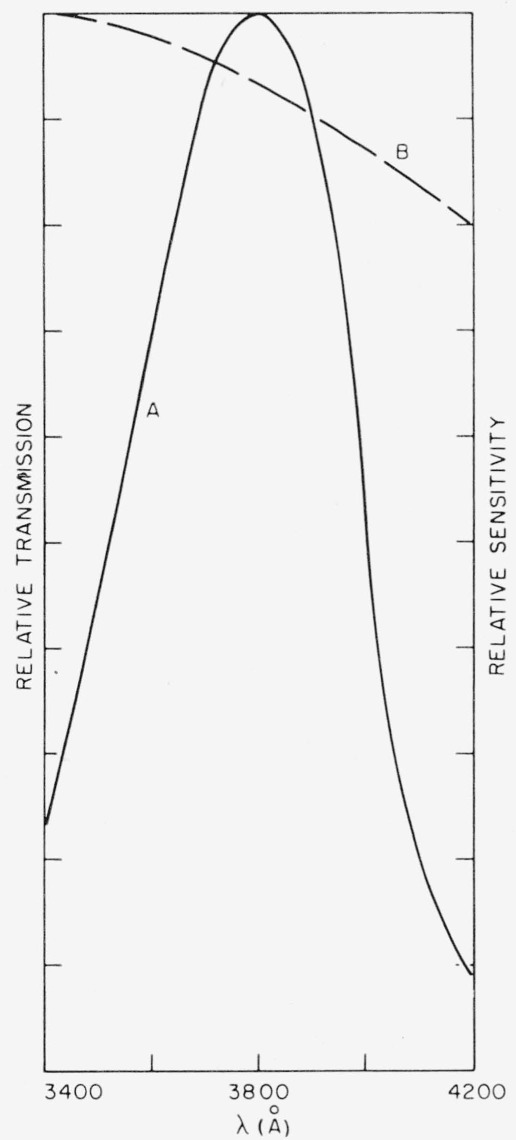
Photometer spectral response for optical density measurements. A—relative transmission curve of filter. B—relative sensitivity of 1P28, hotoinultiplier detector.

**Figure 17:**
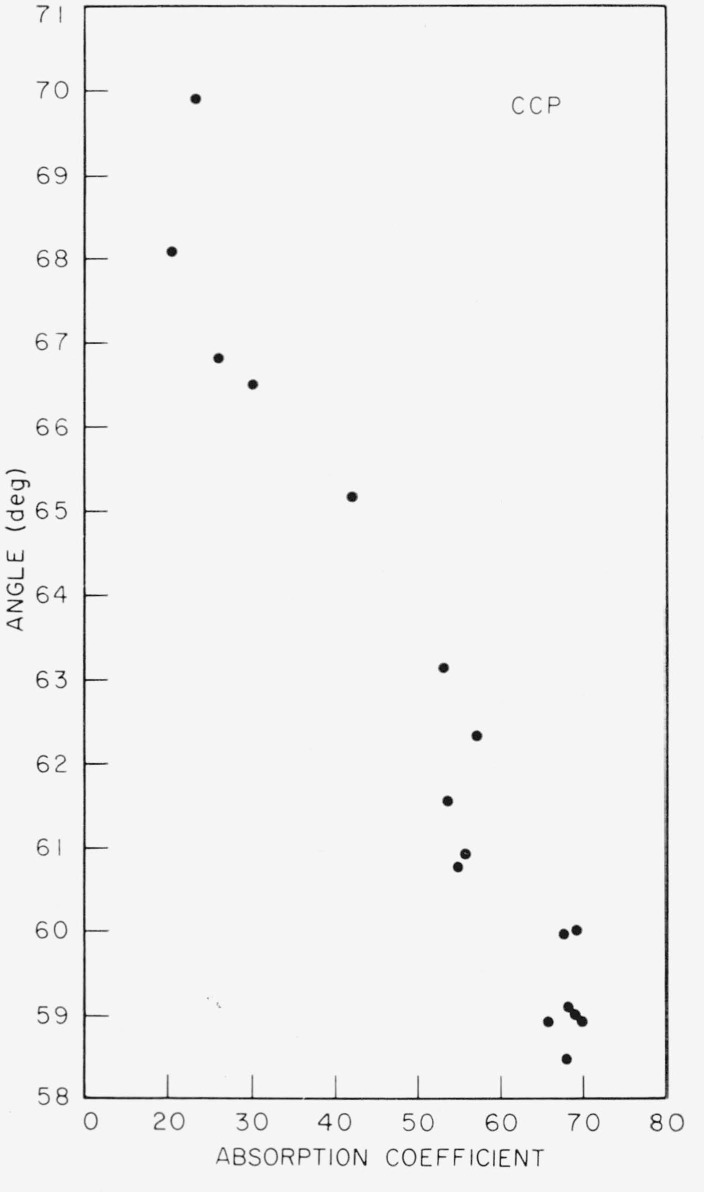
The relationship of apparent optic axial angle to relative absorption coefficient for specimens of rum-green Canadian Clear Purdy (see fig. 5).

With reference to figure 4, the one factor which varies in common with the progression along the color series used in figure 15 is specular density, particularly in the region of the band edge. Therefore, being essentially of one hue but of variable shade (see fig. 5), the rum-green Canadian Clear Purdy (item 15 of table 1) was chosen for comparison of angle to specular density. Specimens were measured photometrically with a tungsten source and a deep violet filter which had the spectrophotometrically determined pass band of figure 16. This filter was chosen to give a measure of the height of the absorption curve in the vicinity of 0.4*μ*. The correlation obtained for angle to density is as shown in figure 17. Whereas the correlation shown in figure 17 is good, no similarly marked correlation was found for the ruby micas.

**Figure 18:**
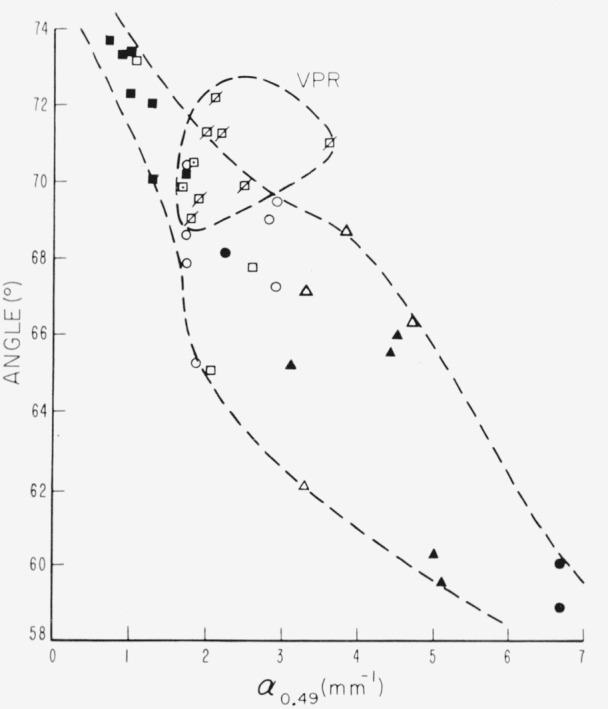
The relationship of apparent optic axial angle to the height of the absorption edge as evaluated by α*_0.49_*. VPR represents the domain of very pink rubies, i.e., α_3.05_ >1mm^−1^ and α_0.58_ >0.2mm^−1^.
☒—very pink ruby□—pink ruby⊡—intermediate α_3.05_○—green rubyΔ—rum■—pale green▲—dark green●—rum-green

A comparison of representatives of all types of the micas, including rubies, showing the relation of apparent axial angle to *α*_0.49_, is seen in figure 18. This presentation tends to clarify the behavior of apparent axial angle. A certain grouping of values occurs for very pink ruby specimens; namely, those specimens for which *α*_3.05_ exceeds 1 mm^−1^ and *α*_0.58_ exceeds 0.2 mm^−1^ all fall within a given domain oí moderately large axial angle and medium *α*_0.49_. The data for all other specimens including several representative of pink rubies, fall in a main sequence in which apparent angle decreases with increasing *α*_0·49_.

It is to be noted that pale green colored specimens, not the ruby, have apparent axial angles approaching that of muscovite proper which ought to be ~78° as obtained from quoted values of index of refraction and axial angle [22].

### 4.5. Absorption Spectra, 10 to 15*μ* of λ

The spectral region from 10 to 15*μ* is interesting from the present viewpoint inasmuch as the cationic molecular vibrations are involved and the relative band intensities show some relation to color.

**Figure 19:**
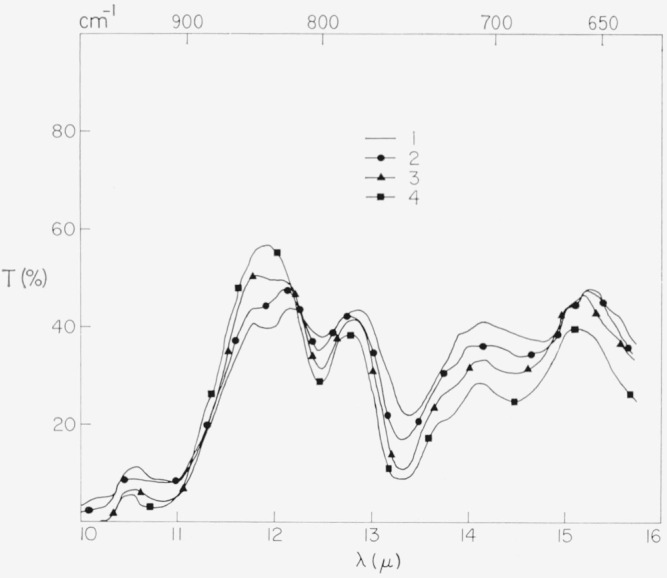
The 10 to 15μ *λ* absorption spectrum and principal color group. 1—*dark green* Tanganyika 6μ thick; 2—*amber* (*rum*) Brazil 8μ thick; 3—*green-white* domestic 7μ thick; 4—*pink ruby* India 8μ thick. Code and associated spectra are those of figures 6 and 7.

Examples of spectral variations found in this *λ* region are indicated in figure 19 for specimens representative of the principal color groups, pink ruby, rum, dark green, and pale green. Generally, transmission increased as the color progressed toward dark green, except in the vicinity of 12*μ* where the opposite was true. The variations of particular band intensities, however, are more involved.

### 4.6. Absorption Coefficient Representation, 10 to 15*μ* of λ

**Figure 20:**
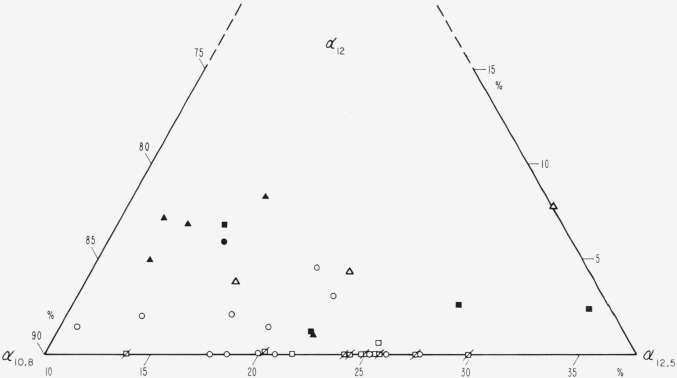
A triangular plot of the relative intensities of the 10.8, 12, and 12.5μ. bands: α*_10.8_*, α*_12_*, α*_12.5_* See figure 18 for code.

The shape of the transmission maximum in the vicinity of 12*μ* is determined by the intensities of the four bands at 10.8, 11.4, 12, and 12.5*μ*. The 11.4*μ* band is too faint for evaluation. A representation which differentiates the absorption coefficient data for the other three bands on the basis of color is given in figure 20. In this the absorption coefficient of each band is normalized by the sum of the coefficients. It is evident that the points shift to the upper left as color progresses from pink ruby to dark green. In detail, pink rubies are characterized by a relatively intense 12.5*μ* hand and by a missing or nearly missing 12*μ* band. Green ruby specimens have a faint 12*μ* band, usually a less intense 12.5*μ* band, but a more intense 10.8*μ* band than the pink ruby specimens. Dark green pieces usually show the greatest 12*μ* absorption and the least 12.5*μ* absorption. Rum and pale green specimens are associated with intermediate 12*μ* absorption.

**Figure 21:**
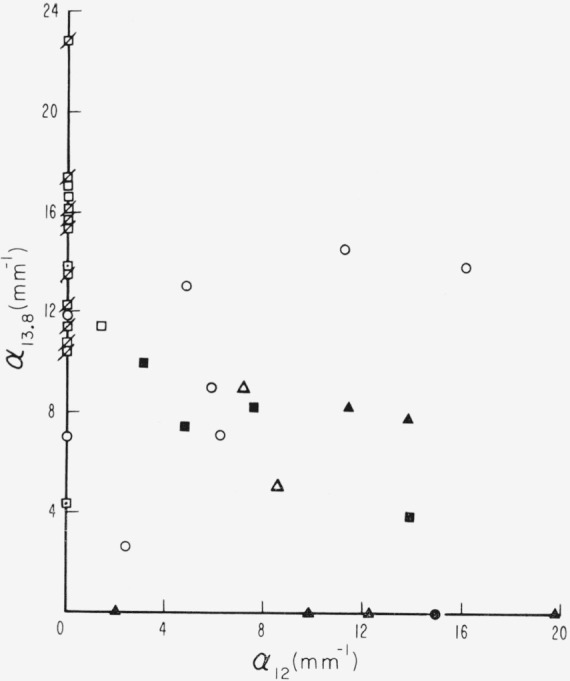
A comparison of the activities of the two weak bands at 12 and 13.8μ for specimens of different color groups. See figure 18 for code.

A second, relatively weak band, at 13.8*μ*, is related to the 12*μ* band. This relationship, similar to that between the 0.44 and 3.05*μ* bands (see fig. 11), is shown in figure 21 in which *α*_13.8_ is plotted against *α*_12_. Although comparatively weak for the 10 to 15*μ* region, these bands at 12 and 13.8*μ* have the same order of intensity as the pink ruby 3.05*μ* band. The pink ruby specimens possess the most intense 13.8*μ* band but little if any 12*μ* band. Dark green specimens are opposite and have least 13.8*μ* absorption but most 12*μ* absorption. Specimens of intermediate color groups show both bands. As lines connecting points to the origin are rotated from the *α*_13.8_ axis to the *α*_12_ axis, color progresses from pink ruby to dark green. Ruby specimens usually appear above a line of slope 1, while nonruby specimens usually appear below this line.

**Figure 22:**
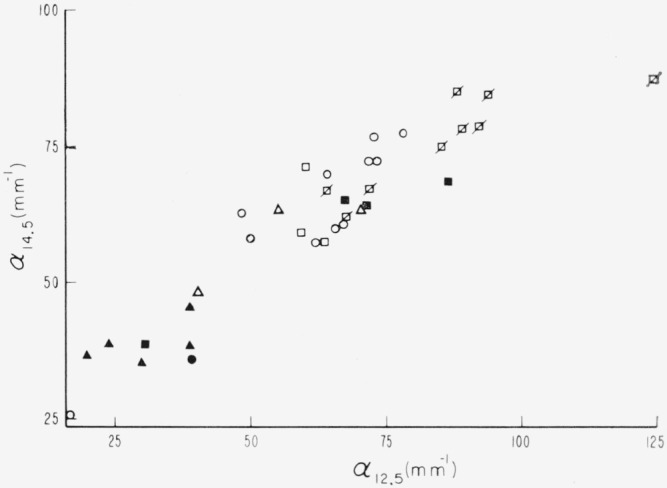
A comparison of the activities of the two strong bands at 12.5 and 14.5μ for specimens of different color groups.

The strengths of the intense bands at 13.3 and at 14.5*μ* are associated with that of the 12.5*μ* band. The increase of the 14.5*μ* band with the 12.5*μ* band is shown in figure 22. As observable from the color code, both *a_i2_* and *α*_14_._5_ increase as color types change from dark green, to rum, to green ruby to very pink ruby.

**Figure 23:**
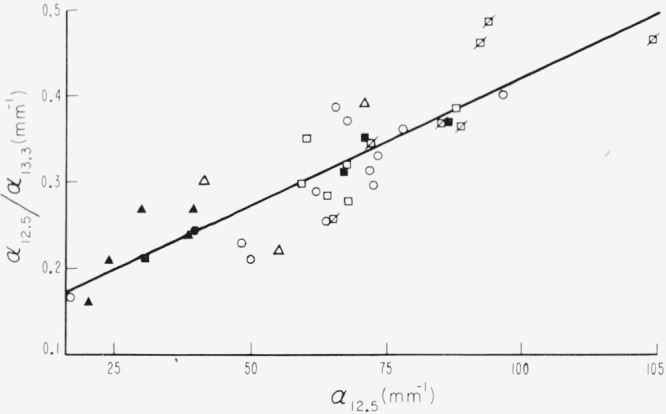
A comparison of the activities of the two strong bands at 12.5 and 13.3μ for specimens of different color groups; α*_12.5_*/α*_13.3_* versus α*_12.5_*.

The strength of the 13.3*μ* band is not linearly related to that of the 12.5*μ* band, but the ratio of the absorption coefficients of the 12.5 and 13.3*μ* bands is somewhat linearly related to *α*_12.5_, as shown in figure 23. A separation of values by color is evident.

No direct correlation was found for any one band in the 10 to 15*μ* region to any one line or band in the shorter wavelength ranges of 0.3 to 1*μ* and 1 to 8*μ*.

## 5. Summary and Concluding Remarks

Muscovite sheet mica has traditionally been categorized primarily as ruby or nonruby, and although physical differences between these two categories have long been debated, no substantial evidence other than color has existed to warrant such differentiation. In the present paper, on the basis of color, apparent optic axial angle, and absorption spectra, we have shown that there are other measurable differences in muscovite mica which provide a more complex categorization than just ruby or nonruby.

The short wavelength absorption spectra (0.3 to 1 *μ* λ) have a deep absorption edge at a wavelength near 0.32*μ* and a number of weak lines and absorption regions at the base of this edge. These spectra can be classified as of three types. All the colors observed in commercial muscovite sheet can be related to variations in these three spectral types. The position of the absorption edge and the activities of weak lines in the visible absorption region sensitively vary with color. These are further related to axial angle and longer wavelength absorption bands. This is interesting, since these lines would be related to energy differences an order of magnitude greater than those directly related to the molecular vibrations. They are reminiscent of those found in a defect structure, e.g., color centers. The position of the absorption edge and the strengths of two weak lines at 0.44 and 0.58*μ* of λ are particularly useful for measuring the variations in these crystals.

The apparent optic axial angle is related to the position of the absorption edge, the angle diminishing as the edge shifts to longer wavelength and lower absorption energy. There are certain ruby specimens for which this relationship is not found. These are particularly absorbant in the blue to yellow region of the visible range and the near infrared at 3 to 3.7*μ*. The values of angle and position of the edge place these ruby materials outside of the main sequence of the other micas.

Ruby micas have an active multiplet in the near infrared between 3 and 3.7*μ* of λ. This multiplet does not appear in other muscovites. The amount of absorption further separates the ruby material into two subclasses.

The spectral bands in the middle infrared between 10 and 15*μ* λ are shown to be related to the color of the material. These bands are in a region which has been shown by others to indicate vibrations of such cationic-oxygen structures as Si—O—Al.

Thus, all in all, it is demonstrated that muscovite sheet micas have measureable differences which by their nature indicate basic chemical and structural variations.

## Figures and Tables

**Figure 24:**
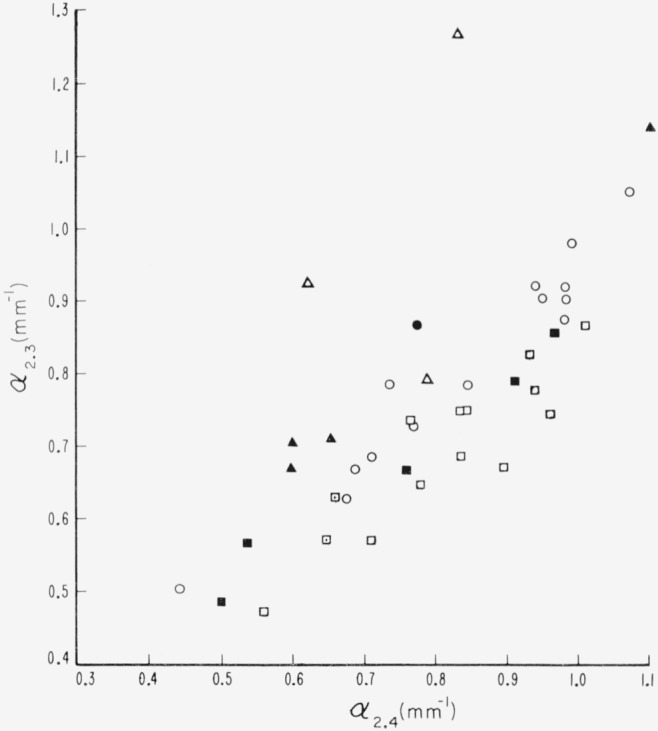
Internal activity of the 2.3 to 2.4μ multiplet with color; α *_2.3_* versus α*_2.4_*_·_

**Figure 25:**
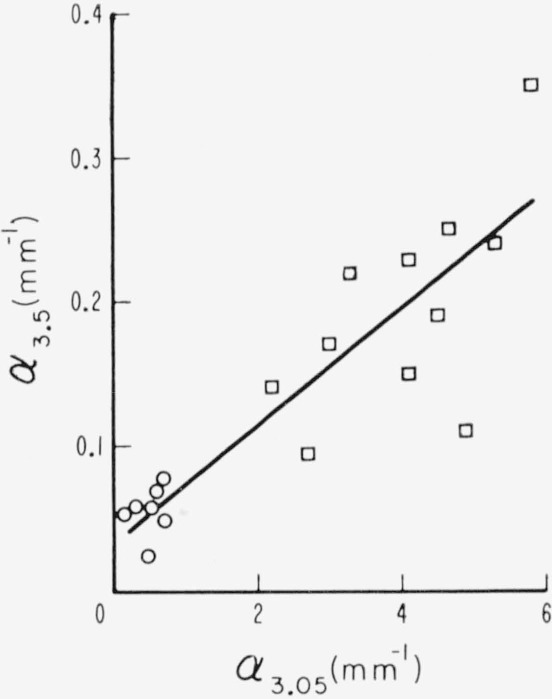
Internal activity of the 3 to 3.5μ multiplet; association of the 3.5μ band to the 3.05μ band.

**Figure 26:**
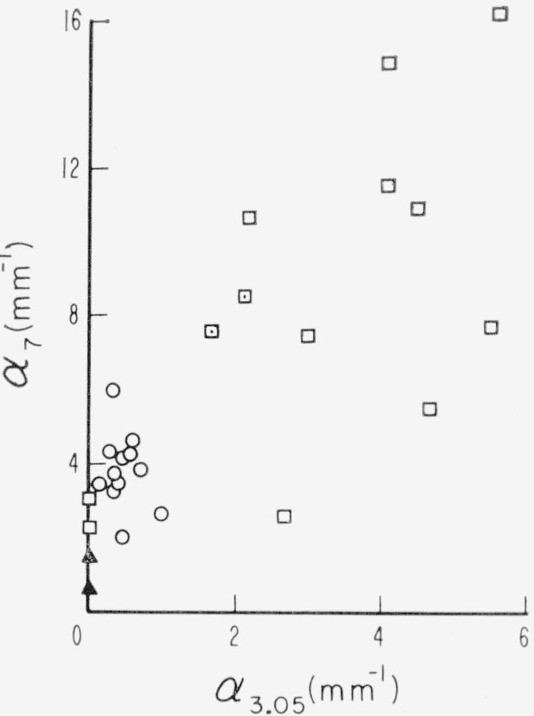
Association of the 7μ band to the 3.05μ band

**Table 1:** Description of muscovite mica used for the study

Color	Origin	Quality	Grade	Amount
				
1. Ruby	India	V–l	5	12 pc.
2. Ruby	India	V–2	6	a5 lb.
3. Ruby	India	V–4	6	5lb.
4. Ruby	India	V–7	6	5 lb.
5. Ruby	United States	V–4	6	5 lb.
6. Ruby	Brazil	V–4	6	5 lb.
7. Ruby, normal good colored.	Brazil	V–l	5	11 pc.
8. Ruby, slightly dark	Brazil	V–l	4 & 5	4 pc.
9. Ruby, medium dark	Brazil	V–l	4 & 5	8 pc.
10. Ruby, very dark (rum ruby).	Brazil	V–l	4,5,5½	4 pc.
11. Ruby^b^	Madras (India)	V–4	6	1 lb.
12. Nonruby	United States	V–4	6	5lb.
13. Amber, rum bordering on ruby.	Brazil	V–l	5	15 pc.
14. Brown or “dark green”	Tanganyika	V–l	4 & 5	15 pc.
15. Clear Purdy	Canada	V–3	4	1lb.
16. Light green	Madras	V–l	5 & 5½	20 pc.
17. Dark green	Madras	V–l	5	15 pc.

a One pound of No. 6 material (here obtained as cleavage plates of 0.15 to 0.5 mm thickness) yielded 500 or more pieces.

b This material was labeled Madras Clear Green but contained many specimens borderline between ruby and nonruby.

c Canadian Clear Purdy. This batch appeared borderline between rum and green.

**Table 2:** Dimensions of grades of mica used in this study

ASTM size	Area of maximum rectangle	Minimum dimension of one side
		
*Grade*	*in*.^2^	*in*.
4	6 up to 10	1½
5	3 up to 6	1
5½	2¼ up to 3	⅞
6	1 up to 2¼	¾

## Footnotes

3 Figures in brackets indicate the literature references at the end of this paper.

## 6. Appendix

Several additional relationships are of interest.

The bands of the 2.2 to 2.5*μ* λ region consist of an absorption spike at 2.2*μ* and a multiplet at 2.3 to 2.4*μ*. These are said to be pleochroic [37]. The 2.3 and 2.4*μ* bands, however, are companions. There is a difference in their intensity ratio with color group (see fig. 24). The 2.4*μ* band is the more intense in pink ruby specimens, but as the color progresses to the other extreme, the 2.3*μ* band becomes stronger. Thus, a trace drawn through the values of the pink rubies in figure 24 is displaced upward on the [*α_2.3_*, α_2.4_] plane as the color of specimens progresses to dark green and rum.

A faint band at 3.5*μ* which is part of the 3 to 3.7*μ* multiplet is comparable in intensity to the faint absorptions in the 0.3 to 1*μ* λ range. Because of its relative weakness, its *a* is not accurately determined, but figure 25 does indicate that the 3.5*μ* band is proportional to the 3.05*μ* band.

The band at 7*μ* is even less accurately evaluated from our spectra (see fig. 1), but it too shows some relationship to the 3.05*μ* band as Sutherland et ah, reported [37], see figure 26.
